# Faster than Virus: The Physics of Pandemic Prediction

**DOI:** 10.3390/idr18010007

**Published:** 2026-01-09

**Authors:** Serena Vita, Giovanni Morlino, Alessandra D’Abramo, Laura Scorzolini, Gaetano Maffongelli, Delia Goletti, Francesco Vairo, Enrico Girardi, Massimo Ciccozzi, Emanuele Nicastri

**Affiliations:** 1 National Institute for Infectious Diseases Lazzaro Spallanzani-IRCCS, Via Portuense 292, 00149 Rome, Italy; serena.vita@inmi.it (S.V.); laura.scorzolini@inmi.it (L.S.); gaetano.maffongelli@inmi.it (G.M.); delia.goletti@inmi.it (D.G.); francesco.vairo@inmi.it (F.V.); enrico.girardi@inmi.it (E.G.); emanuele.nicastri@inmi.it (E.N.); 2 Department of Neuroscience, Mental Health, and Sense Organs, NESMOS, Sapienza University of Rome, 00189 Rome, Italy; 3 INAF/Astronomical Observatory of Arcetri, 50125 Florence, Italy; giovanni.morlino@inaf.it; 4 Unit of Medical Statistics and Molecular Epidemiology, Università Campus Bio-Medico di Roma, 00128 Rome, Italy; m.ciccozzi@unicampus.it

**Keywords:** spillover, preparedness, early detection, pandemic

## Abstract

Background: Zoonotic spillover events with pandemic potential are increasingly associated with environmental change, ecosystem disruption, and intensified human–animal interactions. Although the specific origin and timing of future pandemics remain uncertain, there is a clear need to complement traditional preparedness strategies with approaches that support earlier anticipation and prevention. Objectives: This study aims to propose a conceptual approach to reframe pandemic preparedness toward proactive surveillance and spillover prevention. Methods: We introduce a tachyon-inspired conceptual approach, using a thought experiment based on hypothetical faster-than-light particles to illustrate anticipatory observation of pandemic emergence. The framework is informed by interdisciplinary literature on emerging infectious diseases, One Health surveillance, predictive epidemiology, and public-health preparedness. Results: The proposed approach highlights the importance of proactive, integrated surveillance systems that combine human, animal, and environmental data. Key elements include the use of advanced analytical tools such as neural networks, early characterization of population risk profiles, strengthened public-health infrastructure, coordinated governance, adaptable financial resources, and a resilient healthcare workforce. The integration of animal welfare considerations, translational research, and planetary health principles is emphasized as central to reducing spillover risk. Conclusions: Tachyon-inspired thinking offers a conceptual tool to support a shift from reactive pandemic response toward proactive anticipation and prevention. Embedding integrated surveillance and One Health principles into public-health systems may enhance early detection capacity and contribute to mitigating the impact of future pandemics.

## 1. Introduction

Throughout history, humanity has endured recurrent pandemics caused by a diverse array of infectious agents, including viruses, bacteria, and parasites [1]. From the medieval Black Death, attributed to Yersinia pestis, to the recent Severe Acute Respiratory Syndrome (SARS)-CoV-2 pandemic, these outbreaks have profoundly shaped societies and cultures [2]. Simultaneously, they have spurred advancements in microbiology, epidemiology, and vaccinology [3].

Emerging infectious diseases comprise a heterogeneous group of pathogens. These include previously unidentified agents, as well as existing pathogens expanding their geographic or host range [4,5,6]. Moreover, the resurgence of controlled diseases, the escalation of disease severity, and the emergence of antimicrobial resistance contribute to this complex challenge [1]. In the past three decades, the global emergence of at least forty novel infectious diseases has been documented, underscoring the rapid proliferation of these pathogens and their substantial threat to human health and survival [6]. Notable examples include SARS, H5N1 avian influenza, swine flu (H1N1), Middle East Respiratory Syndrome (MERS), Zika, Ebola, Nipah, and COVID-19 [4].

Given this context, the occurrence of a zoonotic spillover event with pandemic potential is highly probable [7,8,9] specific origin, causative agent, and vulnerable populations remain all uncertain. In evaluating the probability of occurrence and set up early-warning systems an additional layer of complexity that must be considered is pathogen–pathogen interference. Pathogen interference—where infection by one microbe alters susceptibility to or outcomes from another—has been documented across many infectious agents [10,11]. This may involve innate immune activation, resource competition, or modulation by non-coding RNAs (ncRNAs), which can influence host immune responses and disease susceptibility [12,13]. Divergent ncRNA expression profiles have been linked to differential outcomes in viral co-infection, suggesting that such molecular interactions could modify epidemic dynamics and healthcare demand [14]. Incorporating these insights into predictive models and biosurveillance networks could substantially enhance our ability to forecast and mitigate pandemic threats [7]

Proactive efforts in surveillance, prevention, early detection, and preparedness are crucial to mitigate the impact of this future pandemic [15,16,17]. To effectively address this challenge, a significant mental paradigm shift is required [16]. We must engage in a cognitive exercise, we need to mimic the special theory of relativity, travelling through time and space, to shorten the distance between the observers of the phenomenon and the phenomenon itself, the next currently ongoing—but still unrevealed—pandemic. Thus, we will be immediately prepared to catch it at the earliest onset.

## 2. Hypothetical Faster-than-Light Particles: A Thought Experiment

While the fundamental laws of physics currently preclude exceeding the speed of light, a hypothetical faster-than-light (FTL) particle, a tachyon, could theoretically observe the emergence of a future pandemic with perfect clarity, serving as a thought experiment consistent with frameworks used in anticipatory governance and foresight studies [18]. Such a particle would be able to pinpoint the origin of the outbreak, identify the causative microorganism, and potentially gather crucial information to understand and prevent the event entirely. As hypothetical faster-than-light particles (tachyons), we could presumably observe the complete course of a future pandemic with perfect clarity, from the initial zoonotic spillover event to its global spread. This omniscience would allow for the identification of the causative agent and the implementation of preventive measures at the very outset, potentially preventing the pandemic altogether.

Driven by rapid environmental changes, animals will undertake geographic range shifts to ensure their survival [19,20]. This process can facilitate the introduction of parasites and pathogens to new environments, creating novel opportunities for viral exchange among previously allopatric wildlife species resulting in increased virulence among the new host [9]. Furthermore, human expansion into previously uninhabited areas to intensify resource exploitation (e.g., global breeding, hunting, and commercial activities) will further disrupt wildlife communities [19,20]. This increased human–animal interaction can act as a bridge for zoonotic spillover events, potentially triggering the emergence of new outbreaks and pandemics [7,9,20].

Although the laws of physics currently limit faster-than-light travel, the ongoing pandemic demonstrates the constant presence of threats [7].

Climate change and natural disasters are gradually increasing, and with them the possibility that migratory flows of animals and humans may cause radical changes in the ecosystem and geopolitical order. Just as astronomers predict stellar evolution, we can utilize disease surveillance and ecological studies to identify areas with a high risk of emerging infectious diseases. Early identification of these “pandemic hotspots” is crucial for developing preventative measures and rapid responses.

## 3. Translating Tachyons into a Healthcare Context

While tachyons are hypothetical faster-than-light particles in physics, we can conceptually interpret them as tools for anticipating epidemiological events. In practice, this means using advanced surveillance systems, predictive modeling, and real-time ecological and epidemiological data to “observe” the early stages of zoonotic spillover events before they manifest as widespread outbreaks. These tachyon-inspired models help identify high-risk locations, animal hosts, and environmental conditions, enabling proactive measures in public health. Tachyon-inspired surveillance would rely on integrated environmental, veterinary, and human health data, leveraging artificial intelligence and neural networks to detect subtle signals of pathogen emergence [21]. By continuously monitoring wildlife populations, livestock, human cases, and environmental changes, health systems could identify patterns that precede outbreaks, allowing early interventions to prevent a pandemic [20,22].

## 4. Proactive Pandemic Response

What strategies can be implemented to enhance our ability to identify these threats at the earliest possible stage?

Early detection of emerging infectious diseases is paramount for effective containment, as it enables rapid implementation of public health measures, such as isolation, contact tracing, and vaccination, to mitigate disease spread and reduce morbidity and mortality [23,24]. Early detection also facilitates real-time monitoring of disease progression, aiding in the development of effective treatment strategies and informed resource allocation [25]. However, a paradigm shift is necessary [16]. We now require a proactive approach that prioritizes preventing the very first cases from occurring [7,9]. This shift necessarily implies moving beyond preparedness toward true spillover prevention. Preparedness acts only once human cases have appeared, whereas proactive, One Health-based surveillance aims to detect and reduce the ecological and anthropogenic drivers that enable zoonotic transmission in the first place [26,27]. Increasing evidence shows that upstream interventions—targeting deforestation, land-use change, wildlife trade, and livestock–wildlife interfaces—combined with integrated wildlife–livestock–human surveillance systems are both feasible and economically advantageous compared with reactive strategies [27,28,29]. Embedding proactive surveillance within public health planning is therefore essential to lower the probability of spillover and limit the emergence of future pandemics.

A crucial component of this proactive response is the rapid characterization of the age-specific morbidity and mortality profile of the emerging pathogen [30]. Historical pandemics demonstrate distinct age-related vulnerabilities—such as the 1918 influenza disproportionately affecting young adults due to prior immune priming, or COVID-19 primarily impacting older adults and individuals with comorbidities. Recent analyses have shown that SARS-CoV-2 variants exhibited shifting age patterns across successive waves [11,30]. Understanding these profiles early enables more precise deployment of non-pharmaceutical interventions, optimal vaccine prioritization, and accurate capacity planning for pediatric and geriatric care [31]. Integrating age-stratified surveillance into early outbreak investigation is therefore essential for minimizing both health and socioeconomic impacts.

Prevention includes addressing the drivers of disease emergence, namely ecological, meteorological and anthropogenic factors and activities that increase spillover risk, in order to reduce the risk of human infection [29,32]. It is informed by, amongst other actions, biosurveillance in natural hosts, people and the environment, understanding pathogen infection dynamics and implementing intervention activities [33]. To enhance early pandemic response, we propose a multi-pronged approach. Firstly, we advocate for the sustained implementation of public health measures utilizing syndromic systems powered by neural networks [34]. These networks would facilitate real-time communication between frontline healthcare workers (HCWs) and infectious disease, microbiology, and epidemiology units [33]. The neural network should systematically collect and process data, generating actionable insights that can be effectively communicated to healthcare professionals across all levels [35].

We emphasize the need for substantial improvements in public health surveillance capabilities [36]. This requires expanding infrastructure and allocating resources specifically for early detection of emerging infectious diseases. The aim is to develop a sustainable and scalable tool to enhance regional and national health surveillance capacity in preparation for future pandemics.

Furthermore, seamless integration of animal welfare considerations into public health institutions is imperative [37]. Such integration will foster a more comprehensive understanding of zoonotic disease transmission and enable proactive risk mitigation [38].

To accelerate the development of countermeasures against future pandemics, a significant commitment of human and financial resources to translational research is fundamental [39]. Prioritizing novel drug and vaccine platforms with broad-spectrum efficacy is crucial [40]. Additionally, promoting in silico drug modelling can expedite drug discovery and enhance responses to emerging health threats [41]. Streamlined preliminary clinical studies should be supported to bridge the gap between preclinical research and clinical application, expediting the development of potential therapeutic interventions [41].

Secondly, strengthening political governance is critical. A robust governance provides the necessary framework for decision making, resource allocation, and stakeholder engagement [42]. A strong, participatory leadership structure, supported by a clear vision, is indispensable for navigating complex and rapidly evolving challenges [43,44]. This leadership must prioritize open communication, efficient coordination, continuous organizational learning, and a deep understanding of cultural differences [45].

Thirdly, ensuring sufficient and adaptable financial resources is crucial. Comprehensive healthcare financing is essential to ensure equitable access to necessary medical care for all [46]. Rapidly reallocating existing funds is important to address the dynamic nature of pandemics; financial flexibility is vital to prioritize emerging needs and optimize resource allocation across sectors; finally anticipating and preparing for sudden surges in healthcare demands is mandatory indeed financial plans must include contingencies to manage unexpected cost increases and prevent resource shortages [45]. Fourth, a dedicated and well-supported healthcare workforce is fundamental for managing a global pandemic. The rapid increase in patient populations drastically reduces the HCWs to patient ratio thus increasing workload [47]. The complexities and rapid evolution of infectious diseases necessitate a skilled and dedicated team of professionals at all levels [48]. From frontline care providers to public health administrators, healthcare workers play a pivotal role in containing outbreaks, mitigating disease burden, and restoring public health [49]. Moreover, a well-supported HCWs is crucial for maintaining public trust and confidence in health systems. By investing in the education, training, and well-being of healthcare workers, governments and institutions can build a resilient workforce capable of responding effectively to future health crises [50,51]. While technological advancements, such as artificial intelligence, offer promising tools for disease surveillance, prediction, and resource allocation, they cannot replace the critical thinking, clinical judgement, and interpersonal skills of human healthcare professionals [52]. As Adadi [52] aptly points out, a deep understanding of medical and human sciences remains important for guiding us through the territory of pandemic crises. Human empathy, adaptability, and decision-making in complex situations are essential for quality care and cannot be entirely replaced by technology [27]. Policy responses to pandemics must carefully balance direct infection-control benefits with broader societal impacts. Comparative analyses of countries adopting different strategies—such as Sweden’s focus on protecting high-risk groups without full lockdowns—illustrate the complexity of these trade-offs [53]. Early mortality patterns varied across nations and time periods, emphasizing that outcomes depend on multiple contextual factors including population structure, healthcare capacity, and timing of interventions [54]. Evidence-based, adaptive policies that protect vulnerable groups while minimizing long-term social and economic disruption should therefore guide future responses. Beyond infection control, prolonged lockdowns and mobility restrictions have had measurable social and economic costs. Systematic reviews have reported increases in anxiety, depression, and developmental difficulties among children, alongside educational losses, and delayed healthcare access [41,55]. The financial burden of lockdowns, with large national debts incurred to sustain economic inactivity, may also restrict future access to healthcare and contribute to cumulative excess mortality [56]. Preparedness planning should therefore integrate mental-health support, educational continuity, and economic-resilience measures to balance epidemiological benefits with wider societal well-being [57]. Another consideration concerns possible nonspecific, or off-target, effects of vaccines. Several observational studies have explored whether certain vaccines might influence immune responses or all-cause mortality beyond their intended targets [58,59]. For instance, analyses by Benn and colleagues proposed that COVID-19 vaccines could exhibit nonspecific effects on overall mortality [58]. However, these observations remain debated and limited by potential confounding factors. Continuous post-licensure pharmacovigilance and transparent data reporting are essential to clarify such associations and ensure the highest safety standards in future pandemic preparedness [60]. Individual risk factors, including genetic predisposition and obesity, significantly influence COVID-19 morbidity and mortality. Genome-wide association studies have identified specific genetic variants associated with severe COVID-19 outcomes [61]. Obesity has been consistently linked to increased risk of hospitalization, intensive care admission, and adverse clinical outcomes [62,63].

## 5. Conclusions

Future pandemics are inevitable, which underscores the need for proactive, coordinated, and prevention-oriented strategies. Ensuring planetary health—grounded in the interdependence between human well-being and ecological stability—is essential for reducing spillover risk and strengthening global resilience.

Tachyon-inspired thinking provides a conceptual framework for anticipating emergent threats through integrated surveillance, interdisciplinary collaboration, and rapid response systems. Embedding these principles into predictive epidemiology and early-warning infrastructures can enhance timely interventions and improve preparedness. Translating scientific insights into coordinated public-health actions will be crucial for mitigating the impact of future pandemics and safeguarding both human and environmental health.

## Data Availability

Not applicable.
